# Remarks on Elastic Bandages

**Published:** 1788

**Authors:** James Lucas

**Affiliations:** one of the Surgeons of the General Infirmary at Leeds


III. *Remarks on elastic Bandages. Communicated in a Letter to Dr.* Simmons *by Mr.* James Lucas, *one of the Surgeons of the General Infirmary at Leeds.*

HAVING feen a piece of mechanifm, in which circular brafs wires (fimilar to thofe in Vanbutchel's hatbands, &c.) were with ingenuity employed to act in oppofite directions, it immediately occurred to me, that a bandage, with fuch elaftic fprings, might, in many cafes, prove ufeful. Not long after this I had the misfortune to fprain my ancle, and the confinement this accident occafioned gave me an opportunity of paying more attention to the fubject than I otherwife fhould have done, and of contriving a fling with elaftic wires, which enabled me to walk fooner, and with lefs pain, as well as with lefs hazard of a frefh ftrain.

The conftruction of an elaftic bandage is fimple; not expenfive, or burdenfome; but is eafily adapted, readily altered, and capable of being applied to a variety of purpofes.

By communicating my experience on this fubject to different furgeons of my acquaintance, I found that the fame idea had occurred to to Mr. Park, of Liverpool, who ſhewed me ſome ſtrong circular wire, which he had occaſionally uſed in bandages.

The pinmakers have a ready method of ſpinning circular braſs wire; but if it ſhould be required of a flat form, the wire, I am told, muſt be of another ſort, as braſs wire cannot be ſo formed.

The ſtrength of the ſprings is beſt increaſed by an additional number of wires placed near together. Wire, with its circular turns at a little diſtance, is better ſuited for ſuch purpoſes, from its being more readily put in motion, and ſpringing more freely, than that which is ſpun cloſer. Short pieces of the wire will, in general, be preferable to long ones.

I have commonly uſed pieces of circular braſs wire from half an inch to an inch in circumference, and three or four inches long, turned, at each end, into the form of a ring, which may be attached to the middle or any parts of a bandage, belt of cotton, girth web, or leather ſtrap, that part to which the ſprings are faſtened being left ſo looſe as to permit them to act with freedom, and yet not to be overſtretched.

Should

Should one ſpring, or even all the ſprings break, the bandage would ſtill prove a ſufficient ſupport.

Where wire cannot be adopted, probably the vegetable gum, catgut, or ſome other elaſtic ſubſtance, might be ſubſtituted.

Flannel, not only from its being leſs uncomfortable when wet, but alſo from the convenience attending its elaſticity, is now generally preferred to linen for common bandages.

If an elaſtic bandage is ſufficiently ſtrong, it has greatly the advantage of ſtiff metal, or a metal joint, by not, in any reſpect, impeding the natural motion of a joint from being gradually reſtored.

Springs are moſt powerful when contrived to counteract each other, in imitation of the flexor and extenſor muſcles; but the ſprings ſhould be made ſtronger on that part where the ſupport is chiefly required.

It may not only be neceſſary to apply an elaſtic bandage to the affected, but alſo to the neighbouring parts, that the limb may be leſs burdenſome, and that the aid of muſcular powers, unimpaired, may be called in.

It is often requiſite to confine the motion of the ſprings by circular ſtraps, fixed near that part part of the bandage where the different wires are attached, and where the preſſure from the wires would be uneaſy to defend the parts with a quilted pad.

Spring ſtraps, I have no doubt, will often be found of advantage to thoſe who wear artificial legs, or other pieces of mechaniſm, and ſpring belts would, no doubt, greatly aſſiſt thoſe who carry heavy burdens.

When the patella has been fractured, ſuch a contrivance ſeems well calculated to ſupport the knee; and as it is a well-known fact, that the other knee ſeldom long eſcapes the ſame accident, might it not be well to wear an elaſtic bandage on the ſound as well as the injured knee? In a caſe where the ligament of the knee was partially ruptured, a patient of mine was much benefited by the uſe of ſuch a bandage.

In another caſe, where both thighs had been fractured, and the patient remained unable to move for five or ſix months, much more advantage aroſe from the uſe of elaſtic bandages than I could poſſibly have expected, as ſhe had the uſe of both limbs in a ſhort ſpace of time, and can now walk with a ſtick.

As thefe hints may prove of advantage to thofe who have a mechanical turn, I have taken the liberty of fubmitting them to you for publication in the next volume of the Medical Journal.

